# Two new *Leptobrachella* species (Anura, Megophryidae) from the Yunnan-Guizhou Plateau, southwestern China

**DOI:** 10.3897/zookeys.995.55939

**Published:** 2020-11-18

**Authors:** Jian Wang, Zhi-Tong Lyu, Shuo Qi, Zhao-Chi Zeng, Wen-Xiang Zhang, Long-Shan Lu, Ying-Yong Wang

**Affiliations:** 1 State Key Laboratory of Biocontrol / The Museum of Biology, School of Life Sciences, Sun Yat-sen University, Guangzhou 510275, China; 2 Shenzhen Shuanghuamu Biological Technology Co., Ltd, Shenzhen 51800, China; 3 Yunnan Huanglianshan National Nature Reserve, Honghe Hani and Yi Autonomous Prefecture, Yunnan Province 662500, China

**Keywords:** *Leptobrachella aspera* sp. nov., *Leptobrachella dorsospina* sp. nov., molecular phylogeny, morphology, taxonomy

## Abstract

Two new toad species of the genus *Leptobrachella* are described from the Yunnan-Guizhou Plateau of China, based on the combination of molecular and morphological data. The description of *Leptobrachella aspera* Wang, Lyu, Qi & Wang, **sp. nov.** from Huanglianshan Nature Reserve represents the thirteenth *Leptobrachella* species known from Yunnan Province, and the description of *Leptobrachella dorsospina* Wang, Lyu, Qi & Wang, **sp. nov.** from Yushe Forest Park represents the sixth *Leptobrachella* species known from Guizhou Province. These new discoveries further emphasize the extremely high diversity of the *Leptobrachella* toads in these regions.

## Introduction

The generic classifications within the family Megophryidae Bonaparte, 1850 have always been controversial. For example, recent comprehensive approaches have produced different taxonomic schemes for the genus *Megophrys* sensu lato Kuhl and Van Hasselt 1822 (Chen et al. 2017; Mahony et al. 2017; Liu et al. 2018; Li et al. 2020b). The taxonomy of another group of megophrid toads are facing the same problem: Chen et al. (2018) presented the first well-resolved phylogenetic hypothesis for the genera *Leptolalax* Dubois, 1983 and *Leptobrachella* Bonaparte, 1850. They tended towards the most conservative “one-genus option” pending the acquisition of additional data by assigning *Leptolalax* as a junior synonym of *Leptobrachella*. Their results also rejected the hypothesis that *Leptolalax* consists of two subgenera as proposed by Delorme et al. (2006) and Dubois et al. (2010). In this context, the genus *Leptobrachella* currently contains 82 species widely distributed from southern China, west to northeastern India, through Indochina to the island of Borneo (Frost 2020). *Leptobrachella* is a species-rich genus of megophrid frogs, and a large number of new species have been discovered in recent years due to the application of integrative taxonomy incorporating detailed morphological, bioacoustic and molecular analyses (Rowley et al. 2016, 2017; Yang et al. 2016; Yuan et al. 2017; Eto et al. 2018; Nguyen et al. 2018; Wang et al. 2019; Chen et al. 2020; Luo et al. 2020; Qian et al. 2020).

**Figure 1.**
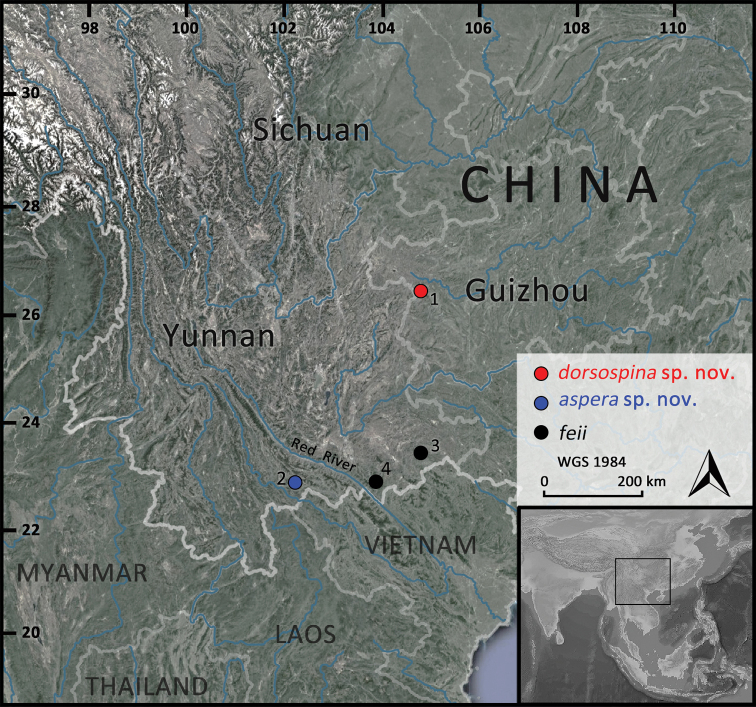
Collection sites. Site 1-Yushe Forest Park, Shuicheng County, Guizhou Province, the type locality of *Leptobrachella dorsospina* sp. nov.; site 2-Huanglianshan Nature Reserve, Lyuchun County, Yunnan Province, the type locality of *L. aspera* sp. nov.; site 3-Xiaoqiaogou Nature Reserve, Xichou County, Yunnan Province, the type locality of *L. feii*; and site 4-Daweishan Nature Reserve, Pingbian County, Yunnan Province, another distribution locality of *L. feii*.

During recent field surveys in the Yunnan-Guizhou Plateau of southwestern China, a number of megophrid specimens were collected from Yushe Forest Park in western Guizhou (Fig. 1, site 1) and Huanglianshan Nature Reserve in southern Yunnan (Fig. 1, site 2), respectively. Morphologically, all the specimens can be assigned to the genus “*Leptolalax*” (now a junior subjective synonym of *Leptobrachella*), based on the following characters: (1) small or moderate size, snout-vent length not greater than 60.0 mm, (2) rounded finger tips, the presence of an elevated inner palmar tubercle not continuous to the thumb, (3) presence of macroglands on body including supra-axillary, pectoral, femoral and ventrolateral glands, (4) vomerine teeth absent, (5) tubercles on eyelids present, and (6) anterior tip of snout with whitish vertical bar (Dubois 1983; Matsui 1997, 2006; Lathrop et al. 1998; Delorme et al. 2006; Das et al. 2010). Although their generic allocation is without doubt, some characters of these specimens do not correspond to the diagnoses of any recognized species. Subsequent molecular analysis further revealed that these specimens represent two distinct evolutionary lineages. Considering both the morphological differences and molecular divergences, these specimens are described herein as two new species.

## Materials and methods

### Sampling

For the molecular analyses, a total of 80 sequences (nine muscle tissue samples was sequenced and 71 sequences obtained from GenBank) were used, including five sequences of the undescribed species from Guizhou, four sequences of the undescribed species from Yunnan, 69 sequences of 66 recognized congeners, and two out-group sequences of *Oreolalax rhodostigmatus* Hu & Fei, 1979 and *Leptobrachium tengchongensis* Yang & Huang, 2019, respectively (Table 1). Due to the presence of cryptic diversity within genus *Leptobrachella*, we chose sequences from type series or topotype specimens for molecular analysis if available to ensure the taxonomic identity of the species being studied.

### DNA Extraction, PCR, and sequencing

DNA was extracted from muscle tissue using a DNA extraction kit from Tiangen Biotech (Beijing) Co., Ltd. The mitochondrial gene 16S ribosomal RNA gene (16S rRNA) fragment from each sample was sequenced. Fragments were ampliﬁed using the primer pairs L3975 (5'-CGCCTGTTTACCAAAAACAT-3') and H4551 (5'-CCGGTCTGAACTCAGATCACGT-3') (Simon et al. 1994). PCR ampliﬁcations were performed in a 20 μl reaction volume with the following cycling conditions: an initial denaturing step at 95 °C for five min; 35 cycles of denaturing at 95 °C for 40 s, annealing at 53 °C for 40 s and extending at 72 °C for one min; and a ﬁnal extending step of 72 °C for 10 min. PCR products were puriﬁed with spin columns. The purified products were sequenced with both forward and reverse primers using BigDye Terminator Cycle Sequencing Kit according to the guidelines of the manufacturer. The products were sequenced on an ABI Prism 3730 automated DNA sequencer in Shanghai Majorbio Bio-pharm Technology Co., Ltd. All sequences have been deposited in GenBank (Table 1).

**Table 1.** Collection localities, voucher data and GenBank numbers (16S rRNA) for all samples used in this study.

ID	Ingroup	Collection Locality	Voucher No.	GenBank No.
**1**	*Leptobrachella aspera* sp. nov.	Huanglianshan Nature Reserve, Lyuchun, Yunnan, China	SYS a007743	MW046199
**2**	*Leptobrachella aspera* sp. nov.	Huanglianshan Nature Reserve, Lyuchun, Yunnan, China	SYS a007744	MW046200
**3**	*Leptobrachella aspera* sp. nov.	Huanglianshan Nature Reserve, Lyuchun, Yunnan, China	SYS a007745	MW046201
**4**	*Leptobrachella aspera* sp. nov.	Huanglianshan Nature Reserve, Lyuchun, Yunnan, China	SYS a007746	MW046202
**5**	*Leptobrachella dorsospina* sp. nov.	Yushe Forest Park, Shuicheng, Guizhou, China	SYS a004961	MW046194
**6**	*Leptobrachella dorsospina* sp. nov.	Yushe Forest Park, Shuicheng, Guizhou, China	SYS a004962	MW046195
**7**	*Leptobrachella dorsospina* sp. nov.	Yushe Forest Park, Shuicheng, Guizhou, China	SYS a004973	MW046196
**8**	*Leptobrachella dorsospina* sp. nov.	Yushe Forest Park, Shuicheng, Guizhou, China	SYS a004974	MW046197
**9**	*Leptobrachella dorsospina* sp. nov.	Yushe Forest Park, Shuicheng, Guizhou, China	SYS a004975	MW046198
**10**	*Leptobrachella feii*	Xiaoqiaogou Nature Reserve, Yunnan, China	KIZ032625	MT302635
**11**	*Leptobrachella feii*	Xiaoqiaogou Nature Reserve, Yunnan, China	KIZ048894	MT302634
**12**	*Leptobrachella feii*	Xiaoqiaogou Nature Reserve, Yunnan, China	KIZ048972	MT302636
**13**	*Leptobrachella feii*	Xiaoqiaogou Nature Reserve, Yunnan, China	KIZ048973	MT302637
**14**	*Leptobrachella aerea*	U Bo, Quang Binh, Vietnam	ZFMK 86362	JN848409
**15**	*Leptobrachella alpina*	Huangcaoling, Jingdong, Yunnan, China	KIZ046816	MH055866
**16**	*Leptobrachella applebyi*	Ngoc Linh, Kon Tum, Vietnam	AMS R 173778	KR018108
**17**	*Leptobrachella arayai*	Mesilau, Sabah, Malaysia	BORNEENSIS 22931	AB847558
**18**	*Leptobrachella ardens*	Kon Ka Kinh, Gia Lai, Vietnam	AMS R 176463	KR018110
**19**	*Leptobrachella bidoupensis*	Hon Giao, Lam Dong, Vietnam	NCSM 77321	HQ902883
**20**	*Leptobrachella bijie*	Zhaozishan Nature Reserve, Guizhou, China	SYS a007320	MK414539
**21**	*Leptobrachella botsfordi*	Fansipan, Lao Cai, Vietnam	AMS R 176540	MH055953
**22**	*Leptobrachella bourreti*	Lao Cai, Vietnam	AMS R 177673	KR018124
**23**	*Leptobrachella chishuiensis*	Chishui, Guizhou Province, China	CIBCS20190518047	MT117053
**24**	*Leptobrachella crocea*	Kon Tum, Vietnam	AMS R 173740	MH055954
**25**	*Leptobrachella dringi*	Gunung Mulu National Park, Sarawak, Malaysia	NMBE1056372	KJ831298
**26**	*Leptobrachella eos*	Long Nai, Phongsaly, Laos	MNHN 2004.0274	JN848452
**27**	*Leptobrachella firthi*	Ngoc Linh Nature Reserve, Kon Tum, Vietnam	AMS R 176524	JQ739206
**28**	*Leptobrachella flaviglandulosa*	Xiaoqiaogou Nature Reserve, Yunnan, China	KIZ032626	MT302633
**29**	*Leptobrachella fritinniens*	Base Camp of Mulu NP, Sarawak, Malaysia	KUHE 55371	AB847557
**30**	*Leptobrachella fuliginosa*	Phetchaburi, Thailand	KUHE 20174	LC201987
**31**	*Leptobrachella gracilis*	Camp 1 of Gunung Mulu NP, Sarawak, Malaysia	NMBE1056364	KJ831300
**32**	*Leptobrachella hamidi*	Bukit Lanjak, Malaysia	KUHE 17545	AB969286
**33**	*Leptobrachella heteropus*	Larut, Malaysia	KUHE 15486	LC202005
**34**	*Leptobrachella isos*	Gia Lai, Vietnam	AMS R 176480	KT824769
**35**	*Leptobrachella kajangensis*	Tioman, Malaysia	LSUHC 4431	LC202001
**36**	*Leptobrachella kalonensis*	Song Luy, Binh Thuan, Vietnam	AMNH A191762	KR018115
**37**	*Leptobrachella kecil*	Cameron, Malaysia	KUHE 52440	LC202004
**38**	*Leptobrachella khasiorum*	Meghalaya, India	SDBDU 2009.329	KY022303
**39**	*Leptobrachella laui*	Tai Mo Shan, Hongkong, China	SYS a002057	KM014546
**40**	*Leptobrachella liui*	Guadun, Mt. Wuyi, Fujian, China	SYS a002479	MH605574
**41**	*Leptobrachella macrops*	Phu Yen, Vietnam	PYU DTD-508	MG787991
**42**	*Leptobrachella maculosa*	Phuoc Binh, Ninh Thuan, Vietnam	ZFMK 96600	KR018120
**43**	*Leptobrachella mangshanensis*	Mangshan Nature Reserve, Hunan, China	MSZTC201701	MG132196
**44**	*Leptobrachella maoershanensis*	Maoershan Nature Reserve, Guangxi, China	KIZ019385	KY986930
**45**	*Leptobrachella marmorata*	Annah Rais, Padawan, Malaysia	KUHE 53192	AB969287
**46**	*Leptobrachella maura*	Kinabalu, Malaysia	SP 21450	AB847559
**47**	*Leptobrachella melanoleuca*	Srat Thani, Thailand	KUHE 19719	LC201990
**48**	*Leptobrachella melica*	Virachey, Ratanakiri, Cambodia	MVZ 258197	HM133599
**49**	*Leptobrachella minima*	Changdao, Thailand	KUHE 23733	LC201980
**50**	*Leptobrachella nahangensis*	Na Hang Nature Reserve, Tuyen Quang, Vietnam	ROM 7035	MH055853
**51**	*Leptobrachella namdongensis*	Thanh Hoa, Vietnam	VNUF A.2017.95	MK965390
**52**	*Leptobrachella niveimontis*	Daxueshan Nature Reserve, Yunnan, China	KIZ015734	MT302618
**53**	*Leptobrachella nyx*	Malipo, Yunnan, China	ROM 35606	MH055814
**54**	*Leptobrachella oshanensis*	Mt. Emei, Sichuan, China	SYS a001830	KM014810
**55**	*Leptobrachella pallida*	Gia Rich, Lam Dong, Vietnam	UNS00510	KR018112
**56**	*Leptobrachella pelodytoides*	Tam Dao, Vinh Phu, Vietnam	MVZ 223642	AY236798
**57**	*Leptobrachella petrops*	Tuyen Quang, Vietnam	VNMN:2016 A.06	KY459998
**58**	*Leptobrachella picta*	Gunung Kinabalu National Park, Sabah, Malaysia	UNIMAS 8705	KJ831295
**59**	*Leptobrachella pluvialis*	Sa Pa, Lao Cai, Vietnam	MNHN: 1999.5675	JN848391
**60**	*Leptobrachella puhoatensis*	Nghe An, Vietnam	AMS R184852	KY849588
**61**	*Leptobrachella purpura*	Yingjiang, Yunnan, China	SYS a006531	MG520355
**62**	*Leptobrachella purpuraventra*	Wujing Nature Reserve, Guizhou, China	SYS a007277	MK414518
**63**	*Leptobrachella pyrrhops*	Loc Bac, Lam Dong, Vietnam	ZMMU ABV-00176	KP017576
**64**	*Leptobrachella rowleyae*	Son Tra, Da Nang, Vietnam	ITBCZ 4113	MG682549
**65**	*Leptobrachella sabahmontana*	Mahua, Crocker, Malaysia	BORNEENSIS 12454	AB847550
**66**	*Leptobrachella shangsiensis*	Shiwandashan, Guangxi, China	NHMG1401032	MK095460
**67**	*Leptobrachella sola*	Terengganu, Malaysia	KUHE 52342	LC202011
**68**	*Leptobrachella suiyangensis*	Suiyang, Guizhou, China	GZNU20180606002	MK829648
**69**	*Leptobrachella sungi*	Bac Giang, Vietnam	ZMMU-NAP-02269	MH055859
**70**	*Leptobrachella tadungensis*	Dak Nong, Vietnam	UNS00517	KR018122
**71**	*Leptobrachella tengchongensis*	Tengchong, Yunnan, China	SYS a004598	KU589209
**72**	*Leptobrachella tuberosa*	Kon Ka Kinh National Park, Gia Lai, Vietnam	ZMMU-NAP-02275	MH055959
**73**	*Leptobrachella ventripunctata*	Xishuangbanna, Yunnan, China	SYS a001768	KM014811
**74**	*Leptobrachella wuhuangmontis*	Mt. Wuhuang, Pubei, Guangxi, China	SYS a003485	MH605577
**75**	*Leptobrachella wulingensis*	Tianzishan Nature Reserve, Hunan, China	CSUFT 200	MT530317
**76**	*Leptobrachella yingjiangensis*	Yingjiang, Yunnan, China	SYS a006533	MG520350
**77**	*Leptobrachella yunkaiensis*	Yunkaishan Nature Reserve, Guangdong, China	SYS a004663	MH605584
**78**	*Leptobrachella zhangyapingi*	Chiang Mai, Thailand	KIZ07258	MH055864
**79**	*Leptobrachium tengchongense*	Tengchong, Yunnan, China	SYS a004603	KX066876
**80**	*Oreolalax rhodostigmatus*	Da Fang, Guizhou, China	CIB ZYCA746	EF397248

### Phylogenetic analyses

Sequences were aligned in Clustal X 2.0 (Thompson et al. 1997) with default parameters. For GenBank sequences which lack information for part of the missing segments, we filled the blank sites with “N”. The aligned data was trimmed allowing no gap positions and default parameters in Gblocks version 0.91b (Castresana 2000). lyWe ran Jmodeltest v2.1.2 (Darriba et al. 2012) with Akaike and Bayesian information criteria on the alignment and obtained the best-fitting nucleotide substitution model of GTR + I + G. Phylogenetic analysis was using Bayesian inference (BI) in MrBayes 3.2.4 (Ronquist et al. 2012). Two independent runs with four Markov Chain Monte Carlo simulations were performed for ten million iterations and sampled every 1000 iterations. The first 25% of samples were discarded as burn-in. Convergence of the Markov Chain Monte Carlo simulations was assessed by PSRF ≤ 0.01 and ESS (effective sample size) value > 200 using Tracer 1.4 (http://tree.bio.ed.ac.uk/software/tracer/). Genetic distances among all *Leptobrachella* samples were calculated in MEGA 6 using the uncorrected *p*-distance model, with pairwise deletion of gaps and missing data.

### Morphometrics

Measurements followed Fei et al. (2009) and Rowley et al. (2013), and were taken with a digital caliper to the nearest 0.1 mm. These measurements were as follows:

**SVL** snout-vent length (from tip of snout to vent);

**HDL** head length (from tip of snout to rear of jaws);

**HDW** head width (head width at commissure of jaws);

**SNT** snout length (from tip of snout to anterior corner of eye);

**EYE** eye diameter (diameter of exposed portion of eyeball);

**IOD** interorbital distance (minimum distance between upper eyelids);

**IND** internasal distance (distance between nares);

**TMP** tympanum diameter (horizontal diameter of tympanum);

**TEY** tympanum-eye distance (distance from anterior edge of tympanum to posterior corner of eye);

**TIB** tibia length (distance from knee to heel);

**ML** manus length (distance from tip of third digit to proximal edge of inner palmar tubercle);

**PL** pes length (distance from tip of fourth toe to proximal edge of the inner metatarsal tubercle);

**LAHL** length of lower arm and hand (distance from tip of the third finger to elbow);

**HLL** hindlimb length (distance from tip of fourth toe to vent).

Sex was determined by the presence of internal vocal sac openings, and the presence of eggs in abdomen seen via external inspection.

All specimens were fixed in 10% buffered formalin and later transferred to 70% ethanol for preservation, and deposited at the Museum of Biology, Sun Yat-sen University (**SYS**) and Chengdu Institute of Biology, the Chinese Academy of Sciences (**CIB**), China; tissue samples were preserved in 95% ethanol for molecular studies.

Comparative morphological data of *Leptobrachella* species were obtained from examination of museum specimens (see Appendix 1) and from the references listed in Table 2. Due to the high likelihood of undiagnosed diversity within the genus (Rowley et al. 2016; Yang et al. 2016), where available, we rely on examination of topotypic material and/or original species descriptions.

**Table 2.** Data sources of the 82 currently known species of the genus *Leptobrachella*.

ID	*Leptobrachella* species	Literature
**1**	*L. aerea* (Rowley, Stuart, Richards, Phimmachak & Sivongxay, 2010)	Rowley et al. 2010c
**2**	*L. alpina* (Fei, Ye & Li, 1990)	Fei et al. 2009, 2016
**3**	*L. applebyi* (Rowley & Cao, 2009)	Rowley and Cao 2009
**4**	*L. arayai* (Matsui, 1997)	Matsui 1997
**5**	*L. ardens* (Rowley, Tran, Le, Dau, Peloso, Nguyen, Hoang, Nguyen & Ziegler, 2016)	Rowley et al. 2016
**6**	*L. baluensis* Smith, 1931	Dring 1983; Eto et al. 2016
**7**	*L. bijie* Wang, Li, Li, Chen & Wang, 2019	Wang et al. 2019
**8**	*L. bidoupensis* (Rowley, Le, Tran & Hoang, 2011)	Rowley et al. 2011
**9**	*L. bondangensis* Eto, Matsui, Hamidy, Munir & Iskandar, 2018	Eto et al. 2018
**10**	*L. botsfordi* (Rowley, Dau & Nguyen, 2013)	Rowley et al. 2013
**11**	*L. bourreti* (Dubois, 1983)	Ohler et al. 2011
**12**	*L. brevicrus* Dring, 1983	Dring 1983; Eto et al. 2015
**13**	*L. crocea* (Rowley, Hoang, Le, Dau & Cao, 2010)	Rowley et al. 2010a
**14**	*L. chishuiensis* Li, Liu, Wei & Wang, 2020	Li et al. 2020a
**15**	*L. dringi* (Dubois, 1987)	Inger et al. 1995; Matsui and Dehling 2012
**16**	*L. eos* (Ohler, Wollenberg, Grosjean, Hendrix, Vences, Ziegler & Dubois, 2011)	Ohler et al. 2011
**17**	*L. feii* Chen, Yuan & Che, 2020	Chen et al. 2020
**18**	*L. firthi* (Rowley, Hoang, Dau, Le & Cao, 2012)	Rowley et al. 2012
**19**	*L. fritinniens* (Dehling & Matsui, 2013)	Dehling and Matsui 2013
**20**	*L. fuliginosa* (Matsui, 2006)	Matsui 2006
**21**	*L. flaviglandulosa* Chen, Wang & Che, 2020	Chen et al. 2020
**22**	*L. fusca* Eto, Matsui, Hamidy, Munir & Iskandar, 2018	Eto et al. 2018
**23**	*L. gracilis* (Günther, 1872)	Günther 1872; Dehling 2012b
**24**	*L. hamidi* (Matsui, 1997)	Matsui 1997
**25**	*L. heteropus* (Boulenger, 1900)	Boulenger 1900
**26**	*L. isos* (Rowley, Stuart, Neang, Hoang, Dau, Nguyen & Emmett, 2015)	Rowley et al. 2015a
**27**	*L. itiokai* Eto, Matsui & Nishikawa, 2016	Eto et al. 2016
**28**	*L. juliandringi* Eto, Matsui & Nishikawa, 2015	Eto et al. 2015
**29**	*L. kajangensis* (Grismer, Grismer & Youmans, 2004)	Grismer et al. 2004
**30**	*L. kalonensis* (Rowley, Tran, Le, Dau, Peloso, Nguyen, Hoang, Nguyen & Ziegler, 2016)	Rowley et al. 2016
**31**	*L. kecil* (Matsui, Belabut, Ahmad & Yong, 2009)	Matsui et al. 2009
**32**	*L. khasiorum* (Das, Tron, Rangad & Hooroo, 2010)	Das et al. 2010
**33**	*L. lateralis* (Anderson, 1871)	Anderson 1871; Humtsoe et al. 2008
**34**	*L. laui* (Sung, Yang & Wang, 2014)	Sung et al. 2014
**35**	*L. liui* (Fei & Ye, 1990)	Fei et al. 2009; Sung et al. 2014
**36**	*L. macrops* (Duong, Do, Ngo, Nguyen & Poyarkov, 2018)	Duong et al. 2018
**37**	*L. maculosa* (Rowley, Tran, Le, Dau, Peloso, Nguyen, Hoang, Nguyen & Ziegler, 2016)	Rowley et al. 2016
**38**	*L. mangshanensis* (Hou, Zhang, Hu, Li, Shi, Chen, Mo & Wang, 2018)	Hou et al. 2018
**39**	*L. maoershanensis* (Yuan, Sun, Chen, Rowley & Che, 2017)	Yuan et al. 2017
**40**	*L. marmorata* (Matsui, Zainudin & Nishikawa, 2014)	Matsui et al. 2014b
**41**	*L. maura* (Inger, Lakim, Biun & Yambun, 1997)	Inger et al. 1997
**42**	*L. melanoleuca* (Matsui, 2006)	Matsui 2006
**43**	*L. melica* (Rowley, Stuart, Neang & Emmett, 2010)	Rowley et al. 2010b
**44**	*L. minima* (Taylor, 1962)	Taylor 1962; Ohler et al. 2011
**45**	*L. mjobergi* Smith, 1925	Eto et al. 2015
**46**	*L. nahangensis* (Lathrop, Murphy, Orlov & Ho, 1998)	Lathrop et al. 1998
**47**	*L. natunae* (Günther, 1895)	Günther 1895
**48**	*L. namdongensis* Hoang, Nguyen, Luu, Nguyen & Jiang, 2019	Hoang et al. 2019
**49**	*L. neangi* Stuart & Rowley, 2020	Stuart and Rowley 2020
**50**	*L. niveimontis* Chen, Poyarkov, Yuan & Che, 2020	Chen et al. 2020
**51**	*L. nokrekensis* (Mathew & Sen, 2010)	Mathew and Sen 2010
**52**	*L. nyx* (Ohler, Wollenberg, Grosjean, Hendrix, Vences, Ziegler & Dubois, 2011)	Ohler et al. 2011
**53**	*L. oshanensis* (Liu, 1950)	Fei et al. 2009, 2016
**54**	*L. pallida* (Rowley, Tran, Le, Dau, Peloso, Nguyen, Hoang, Nguyen & Ziegler, 2016)	Rowley et al. 2016
**55**	*L. palmata* Inger & Stuebing, 1992	Inger and Stuebing 1992
**56**	*L. parva* Dring, 1983	Dring 1983
**57**	*L. pelodytoides* (Boulenger, 1893)	Boulenger 1893; Ohler et al. 2011
**58**	*L. petrops* (Rowley, Dau, Hoang, Le, Cutajar & Nguyen, 2017)	Rowley et al. 2017a
**59**	*L. pictua* (Malkmus, 1992)	Malkmus 1992
**60**	*L. platycephala* (Dehling, 2012)	Dehling 2012a
**61**	*L. pluvialis* (Ohler, Marquis, Swan & Grosjean, 2000)	Ohler et al. 2000, 2011
**62**	*L. puhoatensis* (Rowley, Dau & Cao, 2017)	Rowley et al. 2017b
**63**	*L. purpura* (Yang, Zeng & Wang, 2018)	Yang et al. 2018
**64**	*L. purpuraventra* Wang, Li, Li, Chen & Wang, 2019	Wang et al. 2019
**65**	*L. pyrrhops* (Poyarkov, Rowley, Gogoleva, Vassilieva, Galoyan & Orlov, 2015)	Poyarkov et al. 2015
**66**	*L. rowleyae* (Nguyen, Poyarkov, Le, Vo, Ninh, Duong, Murphy & Sang, 2018)	Nguyen et al. 2018
**67**	*L. sabahmontana* (Matsui, Nishikawa & Yambun, 2014)	Matsui et al. 2014a
**68**	*L. serasanae* Dring, 1983	Dring 1983
**69**	*L. shangsiensis* Chen, Liao, Zhou & Mo, 2019	Chen et al. 2019
**70**	*L. sola* (Matsui, 2006)	Matsui 2006
**71**	*L. suiyangensis* Luo, Xiao, Gao & Zhou, 2020	Luo et al. 2020
**72**	*L. sungi* (Lathrop, Murphy, Orlov & Ho, 1998)	Lathrop et al. 1998
**73**	*L. tadungensis* (Rowley, Tran, Le, Dau, Peloso, Nguyen, Hoang, Nguyen & Ziegler, 2016)	Rowley et al. 2016
**74**	*L. tamdil* (Sengupta, Sailo, Lalremsanga, Das & Das, 2010)	Sengupta et al. 2010
**75**	*L. tengchongensis* (Yang, Wang, Chen & Rao, 2016)	Yang et al. 2016
**76**	*L. tuberosa* (Inger, Orlov & Darevsky, 1999)	Inger et al. 1999
**77**	*L. ventripunctata* (Fei, Ye & Li, 1990)	Fei et al. 2009, 2016
**78**	*L. wuhuangmontis* Wang, Yang & Wang, 2018	Wang et al. 2018
**79**	*L. wulingensis* Qian, Xia, Cao, Xiao & Yang, 2020	Qian et al. in publication
**80**	*L. yingjiangensis* (Yang, Zeng & Wang, 2018)	Yang et al. 2018
**81**	*L. yunkaiensis* Wang, Li, Lyu & Wang, 2018	Wang et al. 2018
**82**	*L. zhangyapingi* (Jiang, Yan, Suwannapoom, Chomdej & Che, 2013)	Jiang et al. 2013

## Results

**Figure 2.**
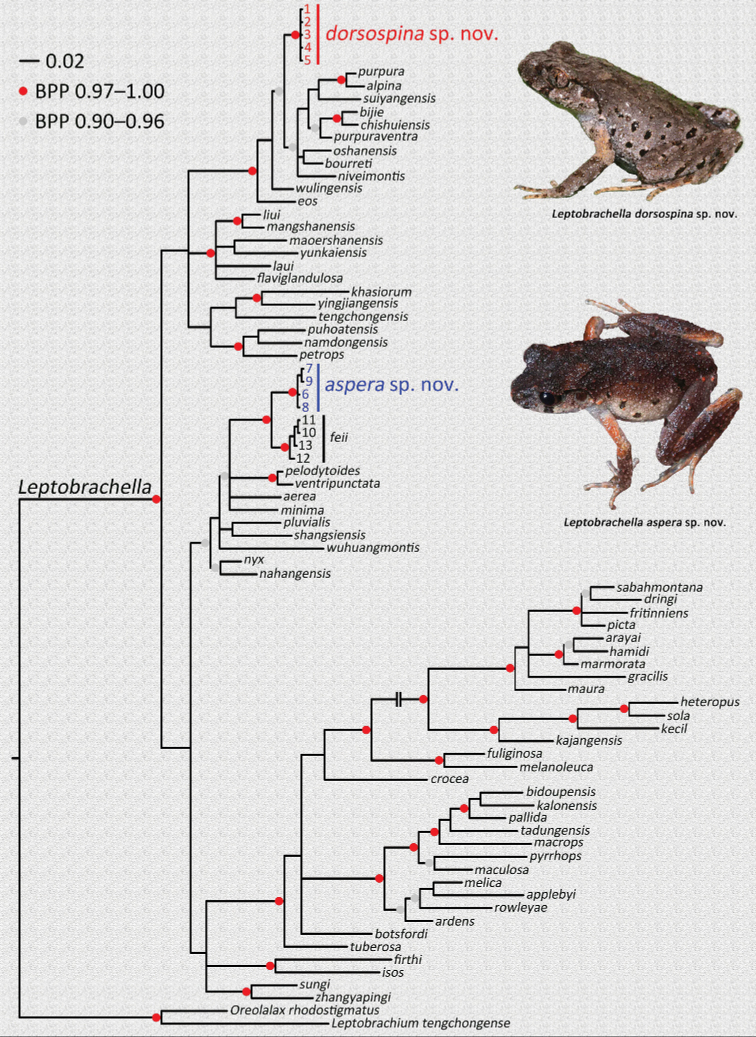
Bayesian Inference tree. The Bayesian posterior probabilities (BPP) > 0.90 were retained.

The BI analyses are shown in Fig. 2 with Bayesian posterior probabilities (BPP) for major nodes > 0.90. Genetic distances among all *Leptobrachella* samples are given in the Suppl. material 1: Table S1. Comparative morphological data of all recognized *Leptobrachella* species occurring north of the Kra Isthmus are listed in Table 3.

As shown by the phylogenetic result, *Leptobrachella* samples from Huanglianshan Nature Reserve are clustered in a distinct and robust monophyletic lineage with strong support (BPP 1.00). This lineage forms the sister taxon to *L. feii* occurring in Xiaoqiaogou Nature Reserve (BPP 1.00). The genetic distances between these two lineages are 3.0–3.4%, which is significantly larger than that among other recognized species (e.g., *p*-distance 2.6% between *L. liui* and *L. mangshanensis*). Detailed morphological examination also reveals a combination of characters that distinguish the specimens of the unnamed lineage from *L. feii* and other known congeners (see taxonomic comparison below). Therefore, based on the molecular and morphological differences, the population from Huanglianshan Nature Reserve is proposed as a new species, *Leptobrachella aspera* sp. nov.

Samples of the other unnamed lineage from Yushe Forest Park, cluster in another distinct and robust monophyletic lineage with strong support (BPP 1.00). This lineage is close to several species occurring in southwestern China, but its specific placement remains unresolved due to the insufficient support values. The smallest genetic distance between this lineage and another congener is 3.5% (vs. *L. purpuraventra*), which is significantly larger than that between other recognized species (e.g., *p*-distance 2.6% between *L. liui* and *L. mangshanensis*). Detailed morphological examination also reveals a combination of characteristics distinguishing the specimens of this lineage from all known congeners (see taxonomic comparison below). Therefore, based on the molecular and morphological differences, the population from Yushe Forest Park is proposed as a new species, *Leptobrachella dorsospina* sp. nov.

**Table 3.** Comparisons of selected diagnostic characters for the new species described herein and congeners occurring north of the Kra Isthmus (modified from Rowley et al. 2017; Wang et al. 2019; Chen et al. 2020).

*Leptobrachella* species	Male SVL (mm)	Black spots on flanks	Toe webbing	Toe fringes	Ventral coloration	Dorsal skin texture
*L. aspera* sp. nov.	22.4	Present	Rudimentary	Narrow	Creamy white with distinct dark patches on chest and abdomen	Rough with dense conical granules, tubercles, and glandular folds
*L. dorsospina* sp. nov.	28.7–30.5	Present	Rudimentary	Narrow	Greyish white with black spots and orange pigmentations	Rough with dense conical granules, tubercles, glandular folds, and conical spines
*L. aerea*	25.1–28.9	Absent	Rudimentary	Wide	Near immaculate creamy white, brown speckles on margins	Finely tuberculate
*L. alpina*	24.0–26.4	Present	Rudimentary	Wide in males	Creamy-white with dark spots	Relatively smooth, some with small warts
*L. applebyi*	19.6–22.3	Present	Rudimentary	Absent	Reddish brown with white speckles	Smooth
*L. ardens*	21.3–24.7	Present	Absent	Absent	Reddish brown with white speckles	Smooth, finely shagreened
*L. bidoupensis*	18.5–25.4	Present	Rudimentary	Weak	Reddish brown with white speckles	Smooth
*L. bijie*	29.0–30.4	Present	Rudimentary	Narrow	White with distinct nebulous greyish speckles on chest and ventrolateral flanks	Shagreened and granular
*L. botsfordi*	29.1–32.6	Absent	Rudimentary	Narrow	Reddish brown with white speckles	Shagreened
*L. bourreti*	28.0–36.2	Present	Rudimentary	Weak	Creamy white	Relatively smooth, some with small warts
*L. crocea*	22.2–27.3	Absent	Rudimentary	Absent	Bright orange	Highly tuberculate
*L. chishuiensis*	30.8–33.4	Present	Rudimentary	Narrow	White with distinct nebulous greyish speckles on chest and ventrolateral flanks	Shagreened and granular
*L. eos*	33.1–34.7	Absent	Rudimentary	Wide	Creamy white	Shagreened
*L. feii*	21.5–22.8	Present	Rudimentary	Narrow	Creamy white with black blotches	Shagreened with small tubercles and ridge
*L. firthi*	26.4–29.2	Absent	Rudimentary	Wide in males	Creamy white	Shagreened with fine tubercles
*L. flaviglandulosa*	23.0–27.0	Present	Poorly developed	Narrow	Whitish with black speckles on margins	Shagreened with yellowish- brown tubercles
*L. fuliginosa*	28.2–30.0	Present	Rudimentary	Weak	White with brown dusting	Nearly smooth with few tubercles
*L. isos*	23.7–27.9	Absent	Rudimentary	Wide in males	Creamy white with white dusting on margins	Mostly smooth, females more tuberculate
*L. kalonensis*	25.8–30.6	Present	Absent	Absent	Pale, speckled brown	Smooth
*L. khasiorum*	24.5–27.3	Present	Rudimentary	Wide	Creamy white	Isolated, scattered tubercles
*L. laui*	24.8–26.7	Present	Rudimentary	Wide	Creamy white with dark brown dusting on margins	Round granular tubercles
*L. liui*	23.0–28.7	Present	Rudimentary	Wide	Creamy white with dark brown spots on chest and margins	Round granular tubercles with glandular folds
*L. lateralis*	26.9–28.3	Present	Rudimentary	Absent	Creamy white	Roughly granular
*L. macrops*	28.0–29.3	Present	Rudimentary	Absent	Greyish violet with white speckles	Roughly granular with larger tubercles
*L. maculosa*	24.2–26.6	Present	Absent	Absent	Brown with few white speckles	Mostly smooth
*L. mangshanensis*	22.2–27.8	Present	Rudimentary	Weak	White speckles on throat and belly	Nearly smooth
*L. maoershanensis*	25.2–30.4	Present	Rudimentary	Narrow	Creamy white chest and belly with irregular black spots	With longitudinal folds
*L. melica*	19.5–22.7	Present	Rudimentary	Absent	Reddish brown with white speckles	Smooth
*L. minima*	25.7–31.4	Present	Rudimentary	Absent	Creamy white	Smooth
*L. nahangensis*	40.8	Present	Rudimentary	Absent	Creamy white with light speckles on throat and chest	Smooth
*L. niveimontis*	22.5–23.6	Present	Rudimentary	Narrow	Marbling with black speckles	Relatively smooth with small tubercles
*L. nokrekensis*	26.0–33.0	Present	Rudimentary	Unknown	Creamy white	Tubercles and longitudinal folds
*L. nyx*	26.7–32.6	Present	Rudimentary	Absent	Creamy white with brown margins	Rounded tubercles
*L. namdongensis*	30.9	Present	Rudimentary	Absent	Immaculate white, chest and belly with dark specking on outer margins	Low, round tubercles, more dense in posterior part of the back
*L. neangi*	-	Present	Weak (in females)	Absent (in females)	Light purplish gray with dark brown mottling on throat	Small, irregular bumps and ridges
*L. oshanensis*	26.6–30.7	Present	Absent	Absent	Whitish with no markings or only small, light grey spots	Smooth with few glandular ridges
*L. pallida*	24.5–27.7	Absent	Absent	Absent	Reddish brown with white speckles	Tuberculate
*L. pelodytoides*	27.5–32.3	Present	Wide	Narrow	Whitish	Small, smooth warts
*L. petrops*	23.6–27.6	Absent	Absent	Narrow	Immaculate creamy white	Highly tuberculate
*L. pluvialis*	21.3–22.3	Present	Rudimentary	Absent	Dirty white with dark brown marbling	Smooth, flattened tubercles on flanks
*L. puhoatensis*	24.2–28.1	Present	Rudimentary	Narrow	Reddish brown with white dusting	With longitudinal skin ridges
*L. purpura*	25.0–27.5	Present	Rudimentary	Wide	Dull white with indistinct grey dusting	Shagreen with small tubercles
*L. purpuraventra*	27.3–29.8	Present	Rudimentary	Narrow	Grey purple with distinct nebulous greyish speckles on chest and ventrolateral flanks	Shagreened with granules
*L. pyrrhops*	30.8–34.3	Present	Rudimentary	Absent	Reddish brown with white speckles	Slightly shagreened
*L. rowleyae*	23.4–25.4	Present	Absent	Absent	Pinkish milk-white to light brown chest and belly with numerous white speckles	Smooth with numerous tiny tubercles
*L. suiyangensis*	28.7–29.7	Present	Rudimentary	Narrow	Yellowish creamy-white with marble texture chest and belly or with irregular light brown speckles	Shagreen with small granules
*L. sungi*	48.3–52.7	Absent or small	Wide	Weak	White	Granular
*L. tadungensis*	23.3–28.2	Present	Absent	Absent	Reddish brown with white speckles	Smooth
*L. tamdil*	32.3	Present	Wide	Wide	White	Weakly tuberculate
*L. tengchongensis*	23.9–26.0	Present	Rudimentary	Narrow	White with dark brown blotches	Shagreened with small tubercles
*L. tuberosa*	24.4–29.5	Absent	Rudimentary	Absent	White with small grey spots/streaks	Highly tuberculate
*L. ventripunctata*	25.5–28.0	Present	Rudimentary	Absent	Chest and belly with dark brown spots	Longitudinal skin ridges
*L. wuhuangmontis*	25.6–30.0	Present	Rudimentary	Narrow	Greyish white mixed by tiny white and black dots	Rough, scattered with dense conical tubercles
*L. wulingensis*	22.7–30.5	Present	Rudimentary	Narrow	Translucent creamy white, with distinct or indistinct brown speckles at margins	Shagreened with sparse large warts, some with longitudinal ridges
*L. yingjiangensis*	25.7–27.6	Present	Rudimentary	Wide	Creamy white with dark brown flecks on chest and margins	Shagreened with small tubercles
*L. yunkaiensis*	25.9–29.3	Present	Rudimentary	Wide	Belly pink with distinct or indistinct speckles	Shagreened with short skin ridges and raised warts
*L. zhangyapingi*	45.8–52.5	Absent	Rudimentary	Wide	Creamy-white with brown margins	Mostly smooth with distinct tubercles

### Taxonomic accounts

#### Leptobrachella aspera

Taxon classificationAnimaliaAnuraMegophryidae

Wang, Lyu, Qi & Wang sp. nov.

19439853-C359-5C28-9B4B-02464BA9160D

http://zoobank.org/4919B18E-B0D0-4329-90BF-8AC77280D263

Fig. 3

##### Type material.

***Holotype*.**SYS a007743, adult male, collected by Jian Wang, Yao Li and Yu-Long Li on 31 May 2019 from Huanglianshan Nature Reserve (22.89°N, 102.29°E; ca. 1930 m a.s.l.), Lyuchun County, Yunnan Province, China.

***Paratypes*** (N = 3). Three adult females, SYS a007744–7745, SYS a007746/CIB116080, the same collection data as the holotype.

**Figure 3.**
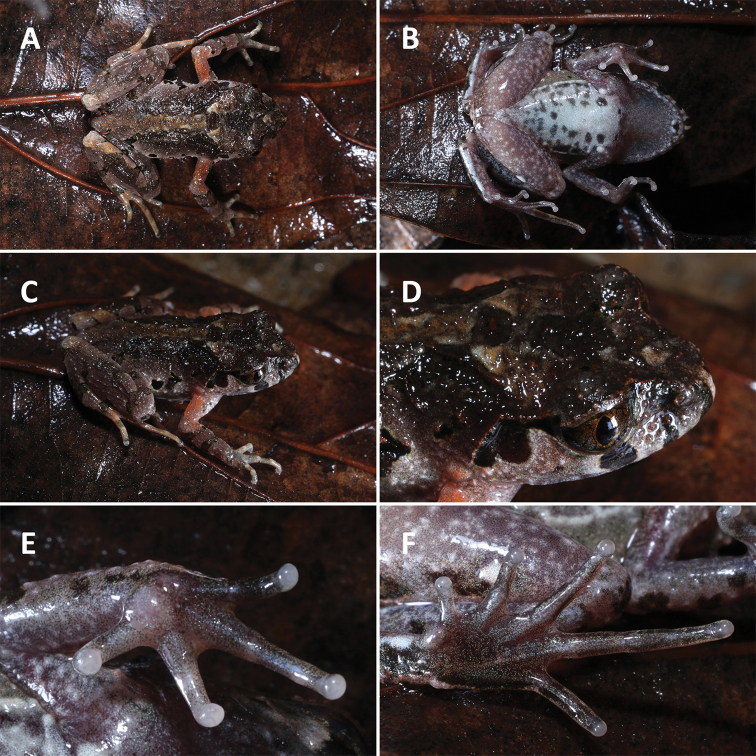
Morphological features in life. *Leptobrachella aspera* sp. nov., holotype SYS a007743.

##### Diagnosis.

(1) Small size (SVL 22.4 mm in a single adult male, 25.0–26.4 in three adult females), (2) dorsal skin rough, with dense conical granules, tubercles and glandular folds, (3) iris bicolored, amber on upper half and silver on lower half, (4) tympanum distinctly discernible, distinct black supratympanic line present, (5) absence of webbing and lateral fringes on fingers, toes with rudimentary webbing and narrow lateral fringes both in males and females, (6) longitudinal ridges under toes not interrupted at the articulations, (7) relative finger lengths I < IV < II < III, relative toe length I < II < V < III < IV, (8) heels just meeting, tibia-tarsal articulation reaches the region between middle of eye to anterior corner of eye, (9) dorsum greyish brown to yellowish brown grounding, with small light orange granules and distinct darker brown markings scattered with irregular light orange or greyish white pigmentations, (10) flanks with several enlarged dark patches with light yellowish green margin, (11) ventral surface creamy white, with distinct regular dark patches on chest and abdomen.

##### Comparison.

From the 26 known congeners of the genus *Leptobrachella* occurring south of the Kra Isthmus, the presence of supra-axillary and ventrolateral glands, can easily distinguish *L. aspera* sp. nov. from *L. arayai*, *L. dringi*, *L. fritinniens*, *L. gracilis*, *L. hamidi*, *L. heteropus*, *L. kajangensis*, *L. kecil*, *L. marmorata*, *L. melanoleuca*, *L. maura*, *L. picta*, *L. platycephala*, *L. sabahmontana* and *L. sola*, all of which lack the supra-axillary and ventrolateral glands; and by the significantly larger body size, SVL 22.4 mm in a single male, *L. aspera* sp. nov. differs from the smaller *L. baluensis* (14.9–15.9 mm in males), *L. brevicrus* (17.1–17.8 mm in males), *L. bondangensis* (17.8 mm in male), *L. fusca* (16.3 mm in male), *L. itiokai* (15.2–16.7 mm in males), *L. juliandringi* (17.0–17.2 mm in males), *L. mjobergi* (15.7–19.0 mm in males), *L. natunae* (17.6 mm in one adult male), *L. parva* (15.0–16.9 mm in males), *L. palmata* (14.4–16.8 mm in males), and *L. serasanae* (16.9 mm in female).

*Leptobrachella aspera* sp. nov. is recovered as a sister taxon to *L. feii* in the phylogenetic tree (Fig. 2). However, the new species can be distinguished from *L. feii* by the following morphological characters: head relatively short, HDL/SVL 0.33–0.35 (vs. head relatively long, HDL/SVL 0.38–0.43); distinct regular dark patches on skin of chest and abdomen (vs. irregular black blotches scattered on skin of chest and belly); color of upper half of iris amber (vs. color of upper half of iris lighter, golden orange); ventrolateral glands forming an non-continuous line (vs. ventrolateral glands forming a continuous line); relative finger lengths I < IV < II < III (vs. relative finger lengths II < I < IV < III); tibio-tarsal articulation of adpressed limb reaching the region between middle of eye to anterior corner of eye (vs. tibio-tarsal articulation of adpressed limb reaching beyond eye).

While *Leptobrachella pluvialis* is distributed in the same mountain range on the Vietnamese side and possesses similar body size (Ohler et al. 2000), it can be separated from *L. aspera* sp. nov. by the following characters: (1) smooth dorsal skin with flattened tubercles on flanks (vs. rough dorsal skin with dense conical granules in *L. aspera* sp. nov.), (2) absence of webbing on toes (vs. rudimentary webbing on toes in *L. aspera* sp. nov.), and (3) relatively longer hindlimbs, the tibia-tarsal articulation reaching to the nostril (vs. relatively shorter hindlimbs, the tibia-tarsal articulation reaching the region between middle of eye to anterior corner of eye in *L. aspera* sp. nov.).

For the remaining 54 members of the genus *Leptobrachella*, in having SVL 22.4 mm in a single male, *L. aspera* sp. nov. differs from the larger *L. aerea* (25.1–28.9 in males), *L. alpina* (24.0–28.9 mm in males), *L. bijie* (29.0–30.4 mm in males), *L. botsfordi* (29.1–32.6 mm in males), *L. bourreti* (28.0–36.2 mm in males), *L. chishuiensis* (30.8–33.4 in males), *L. eos* (33.1–34.7 mm in males), *L. firthi* (26.4–29.2 mm in males), *L. flaviglandulosa* (23.0–27.0 mm in males), *L. fuliginosa* (28.2–30.0 mm in males), *L. isos* (23.7–27.9 mm in males), *L. kalonensis* (25.8–30.6 mm in males), *L. khasiorum* (24.5–27.3 mm in males), *L. laui* (24.8–26.7 mm in males), *L. lateralis* (26.9–28.3 mm in males), *L. macrops* (28.0–29.3 mm in males), *L. maculosa* (24.2–26.6 mm in males), *L. minima* (25.7–31.4 mm in males), *L. nahangensis* (40.8 mm in male), *L. nokrekensis* (26.0–33.0 mm in males), *L. nyx* (26.7–32.6 mm in males), *L. neangi* (30.9 mm in male), *L. namdongensis* (30.9 mm in male), *L. oshanensis* (26.6–30.7 mm in males), *L. pallida* (24.5–27.7 mm in males), *L. pelodytoides* (27.5–32.3 mm in males), *L. petrops* (23.6–27.6 mm in males), *L. puhoatensis* (24.2–28.1 mm in males), *L. purpura* (25.0–27.5 mm in males), *L. purpuraventra* (27.3–29.8 mm in males), *L. pyrrhops* (30.8–34.3 mm in males), *L. rowleyae* (23.4–25.4 mm in males), *L. suiyangensis* (28.7–29.7 mm in males), *L. sungi* (48.3–52.7 mm in males), *L. tadungensis* (23.3–28.2 mm in males), *L. tamdil* (32.3 mm in male), *L. tengchongensis* (23.9–26.0 mm in males), *L. tuberosa* (24.4–29.5 mm in males), *L. ventripunctata* (25.5–28.0 mm in males), *L. wuhuangmontis* (25.6–30.0 mm in males), *L. yingjiangensis* (25.7–27.6 mm in males), *L. yunkaiensis* (25.9–29.3 mm in males), and *L. zhangyapingi* (45.8–52.5 mm in males). By presence of black spots on flanks, the new species can be distinguished from *L. crocea*, versus absence of black spots on flanks; by rudimentary webbing on toes, the new species can be distinguished from *L. ardens*, versus absence of webbing on toes; by narrow lateral fringes on toes, the new species can be distinguished from *L. applebyi*, *L. ardens*, *L. crocea*, and L. melica, all having no lateral fringes on toes, and from *L. liui*, having wide lateral fringes on toes; by the creamy white ventral coloration and distinct regular dark patches on the chest and abdomen, the new species can be distinguished from *L. applebyi*, *L. ardens*, *L. bidoupensis*, and *L. melica*, all having reddish brown ventral coloration with white specks, from *L. crocea*, having bright orange ventral coloration, from *L. mangshanensis*, lacking dark skin patches on the chest and abdomen, from *L. liui*, having creamy white ventral coloration with dark brown spots on the chest and margins, and from *L. niveimontis*, having marbling ventral coloration with black speckling; by rough dorsal skin with dense conical granules, tubercles and glandular folds, the new species can be distinguished from *L. applebyi*, *L. ardens*, *L. bidoupensis*, *L. mangshanensis*, *L. melica*, and *L. niveimontis*, all having smooth dorsal skin.

##### Description of holotype.

Adult male. Body size small, SVL 22.4 mm. Head length slightly larger than head width, HDW/HDL 0.99; snout slightly protruding, projecting slightly beyond margin of the lower jaw; nostril closer to snout than eye; canthus rostralis gently rounded; loreal region slightly concave; interorbital space flat, internarial distance greater than interorbital distance, IND/IOD 1.07; pineal ocellus absent; pupil vertical; snout length longer than eye diameter, SNT/EYE 1.26; tympanum distinct, rounded, and slightly concave, diameter smaller than that of the eye and larger than tympanum-eye distance, TMP/EYE 0.52 and TEY/TMP 0.44; upper margin of tympanum in contact with supratympanic ridge; distinct black supratympanic line present; vomerine teeth absent; vocal sac openings slit-like, paired, located posterolaterally on floor of mouth in close proximity to the margins of the mandible; tongue deeply notched posteriorly; supratympanic ridge distinct, extending from posterior corner of eye to supra-axillary gland.

Tips of fingers rounded, slightly swollen; relative finger lengths I < IV < II < III; nuptial pad absent; subarticular tubercles absent; large, rounded inner palmar tubercle distinctly separated from small, rounded outer palmar tubercle; webbing and lateral fringes on fingers absent. Tips of toes rounded, slightly swollen; relative toe length I < II < V < III < IV; subarticular tubercles absent; distinct longitudinal dermal ridges present under the 3^rd^ to 5^th^ toes, not interrupted; large, oval inner metatarsal tubercle present, outer metatarsal tubercle absent; toes webbing rudimentary; narrow lateral fringes present on all toes. Tibia 47% of snout-vent length; tibiotarsal articulation reaching to anterior corner of eye; heels slightly overlapping when thighs are appressed at right angles with respect to body.

Dorsal skin rough, with dense conical granules, tubercles and glandular folds; ventral skin smooth; sparse tiny tubercles present on surface of chest; pectoral gland and femoral gland oval; the size of pectoral glands almost equal to tips of fingers and femoral glands; femoral gland situated on posteroventral surface of thigh, closer to knee than to vent; supra-axillary glands raised. Ventrolateral glands distinctly visible, raised, forming an incomplete line.

##### Coloration of holotype in life.

Dorsum greyish brown with small light orange granules, distinct darker brown markings scattered with irregular light orange and greyish white pigmentations. A dark brown inverted triangular pattern between the anterior corners of the eyes in connection with a dark brown W-shaped marking in the interorbital region, which is also connected to a W-shaped marking between the axillae. Tympanum dark brown. Small light orange granules present on dorsum of body and limbs; a dark brown blotch under the eye; transverse dark brown bars present on dorsal surface of limbs and digits; distinct dark brown patches with light yellowish green margin on flanks from groin to axilla; elbow and upper arms with distinct coppery orange coloration.

Ventral surface of throat, chest, and belly creamy white; presence of distinct nebulous greyish speckles present on throat, and distinct dark patches on chest and abdomen; ventral surface of limbs greyish purple, scattered with greyish white spots and small patches. Supra-axillary gland coppery orange; femoral, pectoral, and ventrolateral glands greyish white. Iris bicolored, amber on upper half and silver on lower half.

##### Coloration of holotype in preservative

**Figure 4.**
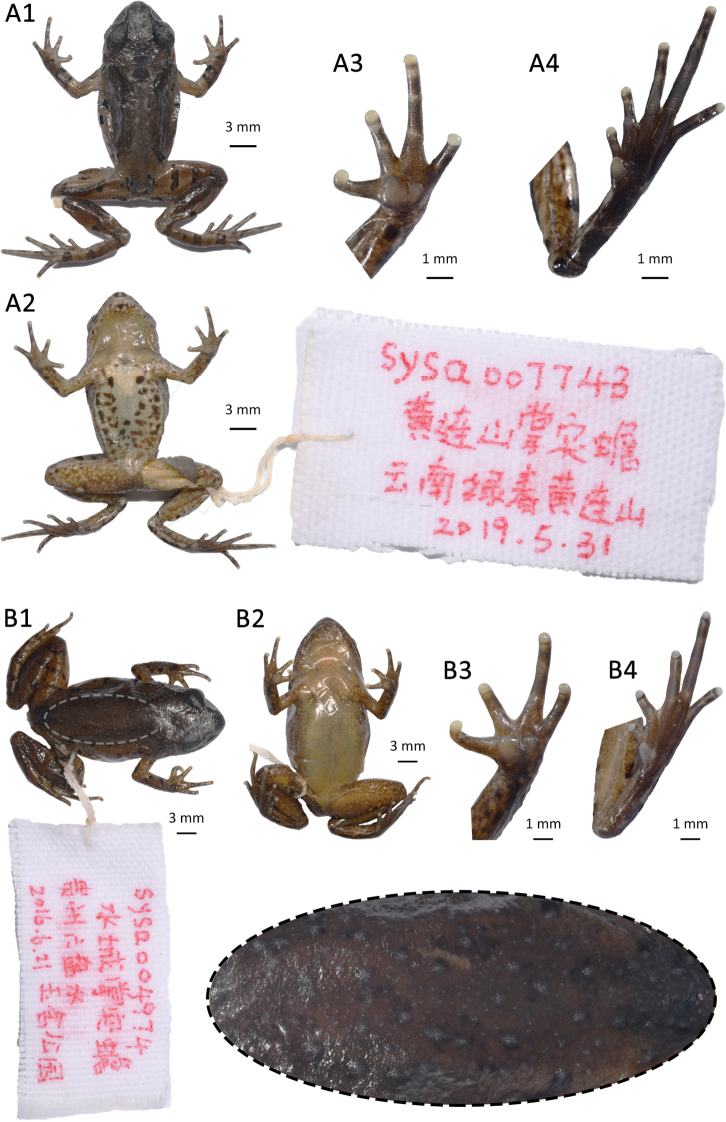
Morphological features in preserved specimens of **A***Leptobrachella aspera* sp. nov., holotype SYS a007743 **B***Leptobrachella dorsospina* sp. nov., holotype SYS a004974. Ellipse selected region showing the tiny spines on dorsal skin.

**(Fig. 4A).** Dorsum of body and limbs dark brown; transverse bars on limbs become more distinct; dark brown patterns, markings and spots on the back become indistinct, orange pigmentations become dark brown, greyish white pigmentations become dark grey. Ventral surface of limbs and surface of throat light brown, surface of abdomen greyish white, nebulous speckles on throat absent, dark patches on chest, abdomen and flanks become more distinct, light yellowish green margin of patches on flanks absent. Supra-axillary, femoral, pectoral, and ventrolateral glands greyish white.

##### Variation.

Measurements and body proportions are listed in Table 4. Nonsexual characters of all the female paratypes (SYS a007744, 7745, 7746) match the overall characters of the holotype except that: the dorsum is greyish brown in the holotype SYS a007743 (vs. yellowish brown in the paratypes); the size of the pectoral glands are almost equal to the tips of the fingers and the femoral glands (vs. the size of the pectoral glands are larger than the tips of fingers and the femoral glands in the paratypes); the tibia-tarsal articulation reaches forward to the anterior corner of the eye in the holotype (vs. the tibia-tarsal articulation reaches forward to the middle of the eye in the paratypes SYS a007745, 7746); the ventral skin of the thighs smooth (vs. the ventral skin of the thighs rough with dense raised tubercles in the paratypes).

**Table 4.** Measurements and body proportions of *Leptobrachella aspera* sp. nov.

Voucher	SYS a 007743	SYS a 007744	SYS a 007745	SYS a 007746
**Sex**	Male	Female	Female	Female
**SVL**	22.4	25.3	25.0	26.4
**HDL**	8.1	9.5	9.5	9.6
**HDW**	8.0	9.3	9.2	9.0
**SNT**	3.7	3.8	3.8	3.4
**IND**	2.5	2.3	2.7	2.7
**IOD**	2.3	2.5	2.5	2.5
**EYE**	2.9	3.2	3.2	3.1
**TMP**	1.5	1.8	1.9	1.6
**TEY**	0.7	1.0	1.0	0.8
**ML**	5.9	7.0	6.6	6.3
**LAHL**	11.2	13.5	12.7	12.6
**PL**	10.1	11.7	10.2	11.1
**TIB**	10.6	12.4	11.9	11.9
**HLL**	34.4	41.5	40.4	39.1
**HDL/SVL**	0.36	0.37	0.38	0.36
**HDW/SVL**	0.36	0.37	0.37	0.34
**HDW/HDL**	0.99	0.98	0.97	0.94
**SNT/HDL**	0.16	0.15	0.15	0.13
**IND/HDW**	0.31	0.25	0.29	0.30
**IOD/HDW**	0.29	0.27	0.27	0.28
**IND/IOD**	1.07	0.91	1.08	1.09
**EYE/HDL**	0.36	0.34	0.34	0.32
**TMP/EYE**	0.52	0.56	0.60	0.51
**ML/SVL**	0.26	0.28	0.26	0.24
**LAHL/SVL**	0.50	0.53	0.51	0.48
**PL/SVL**	0.45	0.46	0.41	0.42
**TIB/SVL**	0.47	0.49	0.48	0.45
**HLL/SVL**	1.53	1.64	1.61	1.48

##### Etymology.

The specific epithet, *aspera*, is a Latin adjective which means rough, in reference to the dorsal skin texture of the new species. According to its type locality, we suggest its English common name as “Huanglianshan Leaf Litter Toad”, and the Chinese name “Huang Lian Shan Zhang Tu Chan (黄连山掌突蟾)”.

##### Distribution and habits.

Currently, *Leptobrachella aspera* sp. nov. is known only from its type locality Huanglianshan Nature Reserve, near the border between China and Vietnam. The new species was found along a drainage ditch of a mountainous road. The road was surrounded by broad-leaved forest at an altitude ca. 1930 m and not close to any hillstreams. Males were not heard calling during the field survey from 31 May to 1 June 2019.

#### Leptobrachella dorsospina

Taxon classificationAnimaliaAnuraMegophryidae

Wang, Lyu, Qi & Wang sp. nov.

F51266DD-BB9E-50C6-A5DB-F2BC1A339F9C

http://zoobank.org/B0EA8FA8-0193-43BF-AA93-6D010467CF84

Fig. 5

##### Type material.

***Holotype*.**SYS a004974, adult male, collected by Zhi-Tong Lyu and Run-Lin Li on 21 June 2016 from Yushe Forest Park (26.47°N, 104.80°E; ca. 2100 m a.s.l.), Shuicheng District, Liupanshui City, Guizhou Province, China.

***Paratypes*** (N = 6). An adult male, SYS a004977, and five adult females, SYS a004961/CIB116081, SYS a 004962, SYS a004973, 4975, 4976, collected by Zhi-Tong Lyu and Run-Lin Li on 20–21 June 2016 from the same locality as the holotype.

**Figure 5.**
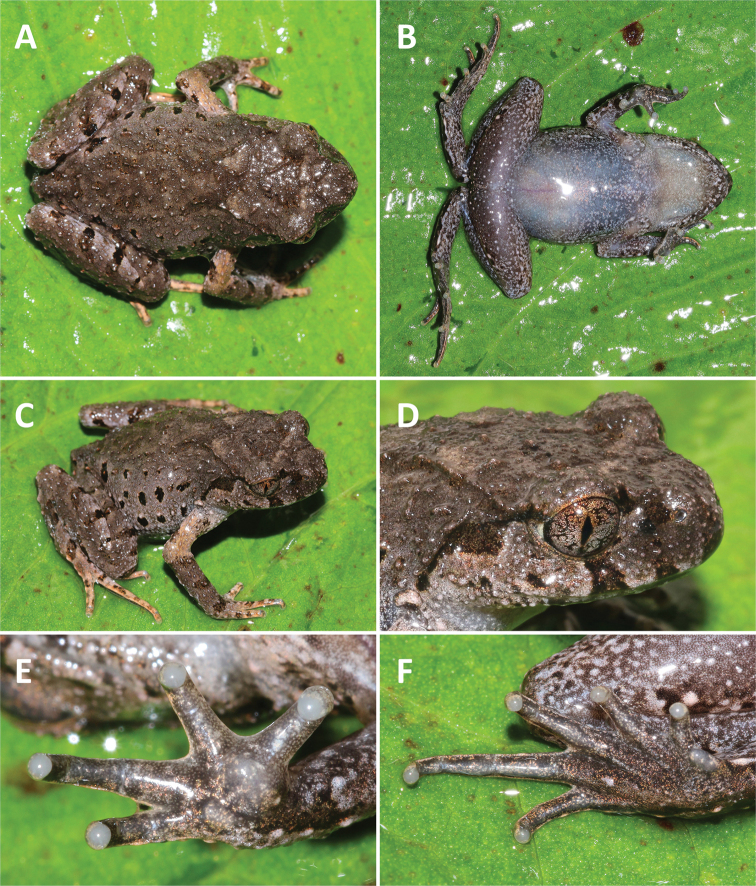
Morphological features in life. *Leptobrachella dorsospina* sp. nov., holotype SYS a004974.

##### Diagnosis.

(1) Small size (SVL 28.7–30.5 mm in two adult males, 32.1–39.8 mm in five adult females), (2) dorsal skin rough, with dense conical granules, tubercles, glandular folds and conical spines, (3) iris bicolored, light orange on upper half and silver on lower half, (4) tympanum distinctly discernible, distinct black supratympanic line present, (5) absence of webbing and lateral fringes on fingers, toes with rudimentary webbing and narrow lateral fringes both in males and females, (6) longitudinal ridges under toes interrupted at the articulations, (7) relative finger lengths II = IV < I < III, relative toe length I < II < V < III < IV, (8) heels slightly overlapping, tibia-tarsal articulation reaches forward to the posterior corners of eyes, (9) dorsum greyish brown to dark brown grounding, with distinct darker brown markings and scattered with irregular light greyish brown pigmentations and yellowish brown spots, (10) flanks with several enlarged dark patches positioned longitudinally in two rows, (11) ventral surface greyish white with black spots and orange pigmentations.

##### Comparison.

Compared with the 26 known congeners of the genus *Leptobrachella* occurring south of the Kra Isthmus, *L. dorsospina* sp. nov. can be easily distinguished by the presence of supra-axillary and ventrolateral glands, from *L. arayai*, *L. dringi*, *L. fritinniens*, *L. gracilis*, *L. hamidi*, *L. heteropus*, *L. kajangensis*, *L. kecil*, *L. marmorata*, *L. melanoleuca*, *L. maura*, *L. picta*, *L. platycephala*, *L. sabahmontana* and *L. sola*, all of which are lacking the supra-axillary and ventrolateral glands; and by the significantly larger body size, SVL 28.7–30.5 mm in two adult male, *L. dorsospina* sp. nov. differs from the smaller *L. baluensis* (14.9–15.9 mm in males), *L. brevicrus* (17.1–17.8 mm in males), *L. bondangensis* (17.8 mm in male), *L. fusca* (16.3 mm in male), *L. itiokai* (15.2–16.7 mm in males), *L. juliandringi* (17.0–17.2 mm in males), *L. mjobergi* (15.7–19.0 mm in males), *L. natunae* (17.6 mm in one adult male), *L. parva* (15.0–16.9 mm in males), *L. palmata* (14.4–16.8 mm in males), and *L. serasanae* (16.9 mm in female).

*Leptobrachella dorsospina* sp. nov. can be easily distinguished from *Leptobrachella aspera* sp. nov. by having distinctly larger body size, SVL 28.7–30.5 mm in males, 32.1–39.8 mm in females (vs. SVL 22.4 mm in male, 25.0–26.4 in females); conical spines on dorsal skin present (vs. absent); black spots on flanks in one row (vs. black spots on flanks in two rows); ventral skin greyish white with black spots and orange pigmentations (vs. ventral skin creamy white with distinct dark patches on chest and abdomen); longitudinal ridges under toes interrupted at the articulations (longitudinal ridges under toes not interrupted at the articulations).

For the remaining 56 members of the genus *Leptobrachella*, in having SVL 28.7–30.5 mm in two males, *L. dorsospina* sp. nov. differs from the larger *L. eos* (33.1–34.7 mm in males), *L. nahangensis* (40.8 mm in male), *L. sungi* (48.3–52.7 mm in males), *L. tamdil* (32.3 mm in male), and *L. zhangyapingi* (45.8–52.5 mm in males); and from the smaller *L. alpina* (24.0–26.4 mm in males), *L. applebyi* (19.6–22.3 mm in males), *L. ardens* (21.3–24.7 mm in males), *L. bidoupensis* (18.5–25.4 mm in males), *L. crocea* (22.2–27.3 mm in males), *L. feii* (21.5–22.8 mm in males), *L. flaviglandulosa* (23.0–27.0 mm in males), *L. isos* (23.7–27.9 mm in males), *L. khasiorum* (24.5–27.3 mm in males), *L. laui* (24.8–26.7 mm in males), *L. maculosa* (24.2–26.6 mm in males), *L. mangshanensis* (22.2–27.8 mm in males), *L. melica* (19.5–22.7 mm in males), *L. niveimontis* (22.5–23.6 mm in males), *L. pallida* (24.5–27.7 mm in males), *L. petrops* (23.6–27.6 mm in males), *L. pluvialis* (21.3–22.3 mm in males), *L. puhoatensis* (24.2–28.1 mm in males), *L. purpura* (25.0–27.5 mm in males), *L. rowleyae* (23.4–25.4 mm in males), *L. tadungensis* (23.3–28.2 mm in males), *L. tengchongensis* (23.9–26.0 mm in males), *L. ventripunctata* (25.5–28.0 mm in males), and *L. yingjiangensis* (25.7–27.6 mm in males). By having black spots on the flanks, *L. dorsospina* sp. nov. can be distinguished from *L. aerea*, *L. botsfordi*, *L. firthi*, and *L. tuberosa*, all of which lack black spots on the flanks. By having rough dorsal skin with conical spines, the new species can be distinguished from *L. bijie*, *L. chishuiensis*, *L. liui*, *L. maoershanensis*, *L. pyrrhops*, *L. purpuraventra*, *L. suiyangensis*, *L. wuhuangmontis*, *L. wulingensis*, and *L. yunkaiensis* (dorsal skin lacking spines); and from *L. bourreti*, *L. fuliginosa*, *L. kalonensis*, *L. minima*, *L. oshanensis*, and *L. pelodytoides* (dorsal skin smooth). By having narrow lateral fringes on the toes, the new species can be distinguished from *L. lateralis*, *L. macrops*, *L. nyx*, *L. pyrrhops*, *L. namdongensis* and *L. neangi*, all of which lack lateral fringes on the toes. The new species can be separated from the remaining *L. nokrekensis* by having greyish white ventral coloration with black patches and orange pigmentations (vs. creamy white), and having dense short glandular folds on the dorsal surface (vs. only a few glandular folds on the dorsal surface).

##### Description of holotype.

Adult male. Body size rather small, SVL 30.5 mm. Head length slightly larger than head width, HDW/HDL 0.99; snout slightly protruding, projecting slightly beyond margin of the lower jaw; nostril closer to snout than eye; canthus rostralis gently rounded; loreal region slightly concave; interorbital space flat, internarial distance smaller than interorbital distance, IND/IOD 0.91; pineal ocellus absent; vertical pupil; snout length larger than eye diameter, SNT/EYE 1.29; tympanum distinct, rounded, and slightly concave, diameter smaller than that of the eye and larger than tympanum-eye distance, TMP/EYE 0.43 and TEY/TMP 0.50; upper margin of tympanum in contact with supratympanic ridge; distinct black supratympanic line present; vomerine teeth absent; vocal sac openings slit-like, paired, located posterolaterally on floor of mouth in close proximity to the margins of the mandible; tongue deeply notched posteriorly; supratympanic ridge distinct, extending from posterior corner of eye to supra-axillary gland.

Tips of fingers rounded, slightly swollen; relative finger lengths II = IV < I < III; nuptial pad absent; subarticular tubercles absent; large, rounded inner palmar tubercle distinctly separated from small, rounded outer palmar tubercle; absence of webbing and lateral fringes on fingers. Tips of toes rounded, slightly swollen; relative toe length I < II < V < III < IV; subarticular tubercles absent; distinct longitudinal dermal ridges present under the 3^rd^ to 5^th^ toes, interrupted; large, oval inner metatarsal tubercle present, outer metatarsal tubercle absent; toes webbing rudimentary; narrow lateral fringes present on all toes. Tibia 44% of snout-vent length; tibiotarsal articulation reaches to posterior corner of eye; heels slightly overlapping when thighs are appressed at right angles with respect to body.

Dorsal skin rough, with dense conical granules, tubercles, glandular folds and conical spines; ventral skin smooth; pectoral gland and femoral gland oval; the size of pectoral glands almost equal to tips of fingers and femoral glands; femoral gland situated on posteroventral surface of thigh, closer to knee than to vent; supra-axillary glands raised. Ventrolateral glands distinctly visible, raised, forming an incomplete line.

##### Coloration of holotype in life.

Dorsum greyish brown with distinct darker brown markings on sides and scattered with irregular light greyish brown pigmentations and yellowish brown spots. An indistinct, darker brown inverted triangular pattern between anterior corners of the eyes, connected to an indistinct dark brown W-shaped marking between the axillae. Dense translucent spines present on dorsal skin of body and limbs. Upper 2/3 of the tympanum dark brown, lower 1/3 light orange, scattered with tiny coppery orange spots. Small greyish white and light brown granules present on the dorsum of the body and limbs; a dark brown vertical bar under the eye; transverse dark brown bars on the dorsal surface of the limbs and digits; distinct dark brown patches on the flanks, from groin to axilla; elbow and upper arms with distinct light orange coloration.

Ventral surface of throat, chest, and belly greyish white; throat with light brown speckles, chest, and abdomen with distinct dark patches; ventral surface of limbs dark grey, scattered with greyish white spots and small patches. Supra-axillary gland light orange; femoral, pectoral, and ventrolateral glands greyish white. Iris bicolored, light orange on upper half and silver on lower half.

##### Coloration of holotype in preservative

**(Fig. 4B).** Dorsum of body and limbs dark brown; transverse bars on limbs, dark brown patterns, markings, and spots on back become indistinct, light greyish brown pigmentations and yellowish spots absent. Translucent spines on dorsal skin of body and limbs become grey. Ventral surface of limbs and surface of throat light brown, surface of abdomen greyish white, dark patches on chest, abdomen and flanks become more distinct. Supra-axillary, femoral, pectoral, and ventrolateral glands greyish white.

##### Variations.

Measurements and body proportions are listed in Table 5. All the female paratypes match the overall characters of the holotype except that: the dorsum is greyish brown in the holotype SYS a004974 (vs. dark brown in the paratypes SYS a004961, 4962), and black spots on the ventral skin are more dense and distinct in the paratypes SYS a004961, 4962.

**Table 5.** Measurements, and body proportions of *Leptobrachella dorsospina* sp. nov.

Voucher	SYS a004977	SYS a004974	SYS a004961	SYS a004962	SYS a004973	SYS a004975	SYS a004976
**Sex**	Male	Male	Female	Female	Female	Female	Female
**SVL**	28.7	30.5	36.1	37.3	39.8	32.1	33.8
**HDL**	10.3	10.8	12.8	12.4	12.9	11.3	12.0
**HDW**	10.6	10.7	11.9	12.9	13.2	11.8	12.2
**SNT**	4.5	4.2	5.1	5.4	5.8	5.0	4.8
**IND**	3.1	3.2	3.6	3.9	4.0	3.7	3.4
**IOD**	3.4	2.9	3.5	3.4	3.3	3.0	2.9
**EYE**	3.5	3.7	3.9	3.7	4.3	4.2	3.8
**TMP**	1.7	1.6	2.3	2.3	2.6	2.1	2.1
**TEY**	1.1	0.8	1.3	1.4	1.5	1.2	1.1
**ML**	7.4	7.3	8.8	7.7	9.1	7.8	7.6
**LAHL**	14.1	14.2	17.1	16.8	17.5	16.2	15.9
**PL**	12.1	12.8	14.9	14.5	15.5	13.9	13.6
**TIB**	13.5	13.4	15.5	16.3	16.6	14.9	14.5
**HLL**	41.7	42.7	49.1	49.9	52.9	46.8	48.0
**HDL/SVL**	0.36	0.35	0.35	0.33	0.32	0.35	0.36
**HDW/SVL**	0.37	0.35	0.33	0.35	0.33	0.37	0.36
**HDW/HDL**	1.03	0.99	0.93	1.04	1.02	1.04	1.02
**SNT/HDL**	0.44	0.39	0.40	0.44	0.45	0.44	0.40
**IND/HDW**	0.29	0.30	0.30	0.30	0.30	0.31	0.28
**IOD/HDW**	0.32	0.27	0.29	0.26	0.25	0.25	0.24
**EYE/HDL**	0.34	0.34	0.30	0.30	0.33	0.37	0.32
**TMP/EYE**	0.49	0.43	0.59	0.62	0.60	0.50	0.55
**ML/SVL**	0.26	0.24	0.24	0.21	0.23	0.24	0.22
**LAHL/SVL**	0.49	0.47	0.47	0.45	0.44	0.50	0.47
**PL/SVL**	0.42	0.42	0.41	0.39	0.39	0.43	0.40
**TIB/SVL**	0.47	0.44	0.43	0.44	0.42	0.46	0.43
**HLL/SVL**	1.45	1.40	1.36	1.34	1.33	1.46	1.42

##### Etymology.

The specific epithet, *dorsospina*, is in reference to the conical spines on the dorsal surface of body in the new species. According to its type locality, we suggest its English common name as “Shuicheng Leaf Litter Toad”, and the Chinese name “Shui Cheng Zhang Tu Chan (水城掌突蟾)”.

##### Distribution and habits.

Currently, *Leptobrachella dorsospina* sp. nov. is known only from its type locality, Yushe Forest Park, which is near the border between Guizhou and Yunnan. The new species was found on the surface of fallen leaves by the clear-water rocky hill-stream in well-preserved montane evergreen broadleaf forest (ca. 2100 m a.s.l.). Males were not heard calling.

## Discussion

In the phylogenetic tree, the *Leptobrachella pelodytoides* (voucher number: MVZ 223642) sample from Tam Dao, northern Vietnam is clustered together with the topotypic *L. ventripunctata* (voucher number: SYS a001768) sample from Xishuangbanna, Yunnan, China, with a genetic divergence of only 1.5% (Fig. 2, Suppl. material 1: Table S1), which is of a intraspecific level. In addition, the type locality of *L. pelodytoides* is Thao [= Thamo], Kayah State, Myanmar, which is geographically distant from northern Vietnam with a distance over 900 km. Considering the above, we recommend that the specimen MVZ 223642 be reappraised as *L. ventripunctata*.

Yunnan and Guizhou are both largely within the species-rich Dian freshwater zoogeographical dominion (Huang et al 2020). Spanning the Indo-Burma Hotspot and the Mountains of Southwest China Hotspot (Tordoff et al. 2012), Yunnan Province has for long been considered as one of the most biodiverse regions in China and its flora and fauna have attracted much attention. However, Guizhou Province, which also shares the Yunnan-Guizhou Plateau, remains relatively neglected; knowledge of biodiversity levels and patterns are seriously lacking. In recent years, large numbers of discoveries have been made from Guizhou, dramatically raising the number of frog species known from the region (Zhang et al. 2017; Li et al. 2018a, b, 2019a, b, 2020a; Lyu et al. 2019; Wang et al. 2019; Luo et al. 2020; Wei et al. 2020). Further comprehensive surveys are urgently needed to determine the true diversity of the amphibians of Guizhou Province.

## Supplementary Material

XML Treatment for Leptobrachella aspera

XML Treatment for Leptobrachella dorsospina

## Appendix 1

**Specimens examined**

***Leptobrachella alpina* (n = 6)**: China: Yunnan Province: Jingdong County: Mt. Wuliang: CIB 24353 (holotype), CIB 24354; SYS a 003927.

***Leptobrachella bijie* (n = 8)**: China: Guizhou: Bijie City: SYS a007313–7320.

***Leptobrachella laui* (n = 26)**: China: Hong Kong: SYS a002057 (holotype), SYS a002058; China: Guangdong Province: Shenzhen City: SYSa 001505–1507, 1515–1521, 3471–3472, 5644–5645.

***Leptobrachella liui* (n = 18)**: China: Fujian Province: Mt. Wuyi: CIB 24355 (holotype), CIB 24356, SYS a001571–1578, 1595–1599, 2478–2479, 5925–5826.

***Leptobrachella mangshanensis* (n = 5)**: China: Guangdong: Nanling Nature Reserve: SYS a002827–2830, 5754.

***Leptobrachella purpuraventra* (n = 15)**: China: Guizhou: Bijie City: SYS a007277–7284, 7300–7306.

***Leptobrachella tengchongensis* (n = 6)**: China: Yunnan Province: Baoshan City: Mt. Gaoligong: SYS a004600 (holotype), 4596–4599, 4601–4602.

***Leptobrachella wuhuangmontis* (n = 12)**: China: Guangxi Province: Pubei County: Mt. Wuhuang: SYS a003500/CIB107274, SYS a000578, 0580–0581, 3485–3489, 3499, 3504–3506.

***Leptobrachella yunkaiensis* (n = 8)**: China: Guangdong Province: Maoming City: Dawuling Forest Station: SYS a004664/CIB107272, SYS a004663, 4665–4669, 4690.

## Supplementary materials

Supplementary material 1 Table S1. Pairwise distances based on 16S gene among all sample used in this study Data type phylogenetic Explanation note Genetic distances among all *Leptobrachella* samples. https://binary.pensoft.net/file/476865This dataset is made available under the Open Database License (http://opendatacommons.org/licenses/odbl/1.0/). The Open Database License (ODbL) is a license agreement intended to allow users to freely share, modify, and use this Dataset while maintaining this same freedom for others, provided that the original source and author(s) are credited.Jian Wang, Zhi-Tong Lyu, Shuo Qi, Zhao-Chi Zeng, Wen-Xiang Zhang, Long-Shan Lu, Ying-Yong Wang
